# A five-year randomized parallel and blinded clinical trial of an extended specialized early intervention vs. regular care in the early phase of psychotic disorders: study protocol

**DOI:** 10.1186/s12888-015-0404-2

**Published:** 2015-02-14

**Authors:** Danyael Lutgens, Srividya Iyer, Ridha Joober, Thomas G Brown, Ross Norman, Eric Latimer, Norbert Schmitz, Amal Abdel Baki, Sherezad Abadi, Ashok Malla

**Affiliations:** Department of Psychiatry, McGill University; Prevention and Early Intervention Program for the Psychoses, Douglas Mental Health University Institute, Montreal, Quebec Canada; Department of Psychiatry, McGill University; Program Coordinator, Prevention and Early Intervention Program for the Psychoses, Douglas Mental Health University Institute, Montreal, Quebec Canada; Department of Psychiatry, McGill University; Assistant Director, Prevention and Early Intervention Program for the Psychoses, Douglas Mental Health University Institute, Montreal, Quebec Canada; Department of Psychiatry, McGill University; Douglas Hospital Research Centre, Montreal, Quebec Canada; Department of Epidemiology and Biostatistics, Western University; Prevention and Early Intervention Program for Psychoses (PEPP), London Health Sciences Centre, South Street Hospital, London, Ontario Canada; Social and Transcultural Division, Department of Psychiatry, McGill, University; Douglas Hospital Research Centre, Montreal, Quebec Canada; Department of Psychiatry & Department of Epidemiology and Biostatistics, McGill, University; Douglas Hospital Research Centre, Montreal, Quebec Canada; Department of Psychiatry, Université de Montréal, Research Centre CHUM, Montréal, QC Canada; Coordinator, Prevention and Early Intervention Program for the Psychoses, Douglas Mental Health University Institute, Montreal, Quebec Canada; Department of Psychiatry, McGill University; Director, Prevention and Early Intervention Program for the Psychoses, Douglas Mental Health University Institute, Montreal, Quebec Canada

**Keywords:** Specialized early intervention, First episode psychosis, Treatment, Critical period, Remission, Case management, Randomized controlled trial

## Abstract

**Background:**

Specialized Early Intervention services (SEI) for first episode psychosis are shown to be effective for the treatment of positive and negative symptoms, medication adherence, rates of relapse, substance abuse disorders, functional outcome and quality of life at two-year treatment follow up. However, it is also reported that these benefits are not maintained when SEI is not sustained. The objective of this trial is to test the efficacy of a 3-year extension of a SEI service (following 2 years of SEI prior to randomization) for the maintenance and consolidation of therapeutic gains as compared to regular care in the community.

**Methods:**

Following an initial 2 years of SEI, patients are randomized to receive either 3-years of continued SEI or regular care. SEI provided at three sites within the McGill network of SEI services, using a model of treatment comprised of: modified assertive case management; psycho education for families; multiple family intervention; cognitive behavioural therapy; and substance abuse treatment and monitoring. Blinded research assistants conduct ongoing evaluation of the outcome variables every three months. The primary outcome measure is remission status measured both as the proportion of patients in complete remission and the mean length of remission achieved following randomization during the additional three years of follow up. Based on preliminary data, it is determined that a total of 212 patients are needed to achieve adequate statistical power. Intent to treat with the last observation carried forward will be the primary method of statistical analysis.

**Discussion:**

The “critical period” hypothesis posits that there is a five year window during which the effects of the nascent psychotic illness can be countered and the impact of the disorder on symptomatic and functional outcomes can be offset through active and sustained treatment. Providing SEI throughout this critical period may solidify the benefits of treatment such that gains may be more sustainable over time as compared to intervention delivered for a shorter period. Findings from this study will have implications for service provision in first episode psychosis.

**Trial registration:**

ISRCTN11889976

## Background

Psychotic disorders including schizophrenia spectrum and affective psychosis are considered among the most severe mental disorders [1], resulting in personal and family suffering [2,3] and associated with poor long term outcomes, particularly if not adequately treated [4-6]. The first episode of psychosis (FEP) typically occurs between adolescence and early adulthood [7] and interferes with educational and employment attainment as well as social transitions [8]. The life time prevalence of all psychotic disorders, that include the presence of at least one positive symptom, is up to 3.4% in the general population [9]. In Canada alone, the overall economic cost to society that includes loss of productivity as a result of psychosis is estimated at over six billion dollars per year [10].

While most patients will respond positively to initial treatment [11], the long-term prognosis is varied [12]. Only up to one quarter of all psychosis patients are likely to achieve complete remission, depending on the criteria and length of follow up applied [12,13]. Given such likely negative outcomes, emphasis has been placed on the earliest phase of psychosis, considered a “critical period” of five years [14] when patients’ psychosocial health may otherwise be most likely to decline [15] unless long term health trajectories are re-established [16]. Specialized Early Intervention (SEI) programs were first initiated in the 1990s as a response to mounting evidence of the importance of prevention, early detection and appropriately targeted and timely treatment during this critical period [17].

In comparison to regular care where the majority of patients (80%) fail to sustain remission within the first five years [18,19], FEP patients treated in an SEI model, show higher rates of remission, lower rates of residual positive and negative symptoms, lowered rates of relapse, less substance abuse and better overall functioning at one and at two years [11,20-24]. Indeed, the benefits of SEI service as compared to treatment as usual over the short term have been verified by three randomized controlled trials [25-28] as well as uncontrolled studies (for a meta-analytic review see Harvey et al., 2007 [29]). Despite such encouraging outcomes, a five year uncontrolled study of FEP patients treated for 12 months in an SEI service and then transferred into regular care showed a loss of the beneficial effects achieved earlier in treatment [30]. Critically, in a five year follow up study (OPUS Trial) of a large sample of FEP patients, who had received two years of SEI service before being transferred to regular care, the therapeutic gains achieved at two years were not maintained over the following three years [31].

This loss of advantage seen over the subsequent three years during which SEI services were no longer available may have been prevented if the SEI service were continued throughout the critical five-year period in FEP. In a recent study conducted in Canada, a reduced level of SEI service was offered to all patients for three years beyond the standard first two years of SEI treatment [32]. Although there was no comparison group and the intensity of SEI was lowered, patient outcome data after five years (two years of SEI followed by three years of stepped down SEI) when compared to the five-year outcome data of OPUS patients who had only received two years of SEI treatment were significantly better (rates of remission and hospitalization) [32].

Based on the evidence reviewed above, the current study is being conducted to address this question of optimum treatment length using a randomized controlled (RCT) design at the Prevention and Early Intervention Program for Psychosis (PEPP-Montreal). In this RCT we evaluate the effect of three years of extension of full SEI services following two years of SEI, compared to three years of regular care following the initial two years of SEI service.

The **primary hypothesis** guiding this RCT is that individuals in the experimental group (extended SEI) will show higher rates and longer periods of remission (both positive and negative symptoms) than the control group over the extension period of three years. The *secondary hypotheses* are that: a) the difference in remission rates are mediated by the level of medication adherence in the two groups; b) as the experimental group is expected to have higher levels of working alliance with their treatment providers than the control group, we hypothesize that the difference in the level of medication adherence between the two groups and retention in treatment is predicted by working alliance; c) that the experimental group will have better clinical outcomes (lower relapse rates and levels of symptoms), functional outcomes (social/occupational functioning), and quality of life than the control group. The economic consequences of extending SEI past the standard current 2 years is also being investigated, within the RCT design, taking into consideration both direct and indirect costs.

## Methods

### Design

This trial is a randomized controlled trial comparing extended SEI for FEP (five years total) with treatment as usual for FEP (two years of SEI followed by three years of regular care). Prior to randomization, all patients received their treatment from the McGill University network of hospitals that offer SEI service according to a common model of care within a defined catchment area in the city of Montreal.

### Inclusion criteria

Our aim is to use inclusion criteria that are as non discriminatory as possible in order to ensure the ecological validity of this trial and to reflect the kinds of diverse patients seen in FEP clinical settings. Although the SEI services treat patients between 14 and 35 years old, for the purpose of the study, patients aged 18–35, with a DSM-IV diagnosis of a psychotic disorder (schizophrenia spectrum psychoses and affective psychosis), who have completed two years of SEI treatment and follow-up within the McGill network of SEI services, an IQ greater than 70, the ability to communicate in either French or English, and the ability to provide informed consent, are eligible for participation. Patients are recruited regardless of their remission status at the end of two years of SEI treatment, consistent with what may be seen in regular clinical practice. Ethics approval for this RCT was granted by McGill University’s Faculty of Medicine Institutional Review Board (Assurance number: FWA 00004545) and from the Douglas Hospital Research Ethics Board.

### Exclusion criteria

Patients who are not able to provide informed consent (as determined by an inability to provide a brief summary of the treatment protocol following presentation of the consent form); those with an inability to communicate in either English or French; and those with an I.Q. below 70 are ineligible for participation. Co-morbid substance abuse and dependence is not an exclusion criteria.

### Randomization

**Figure 1:**
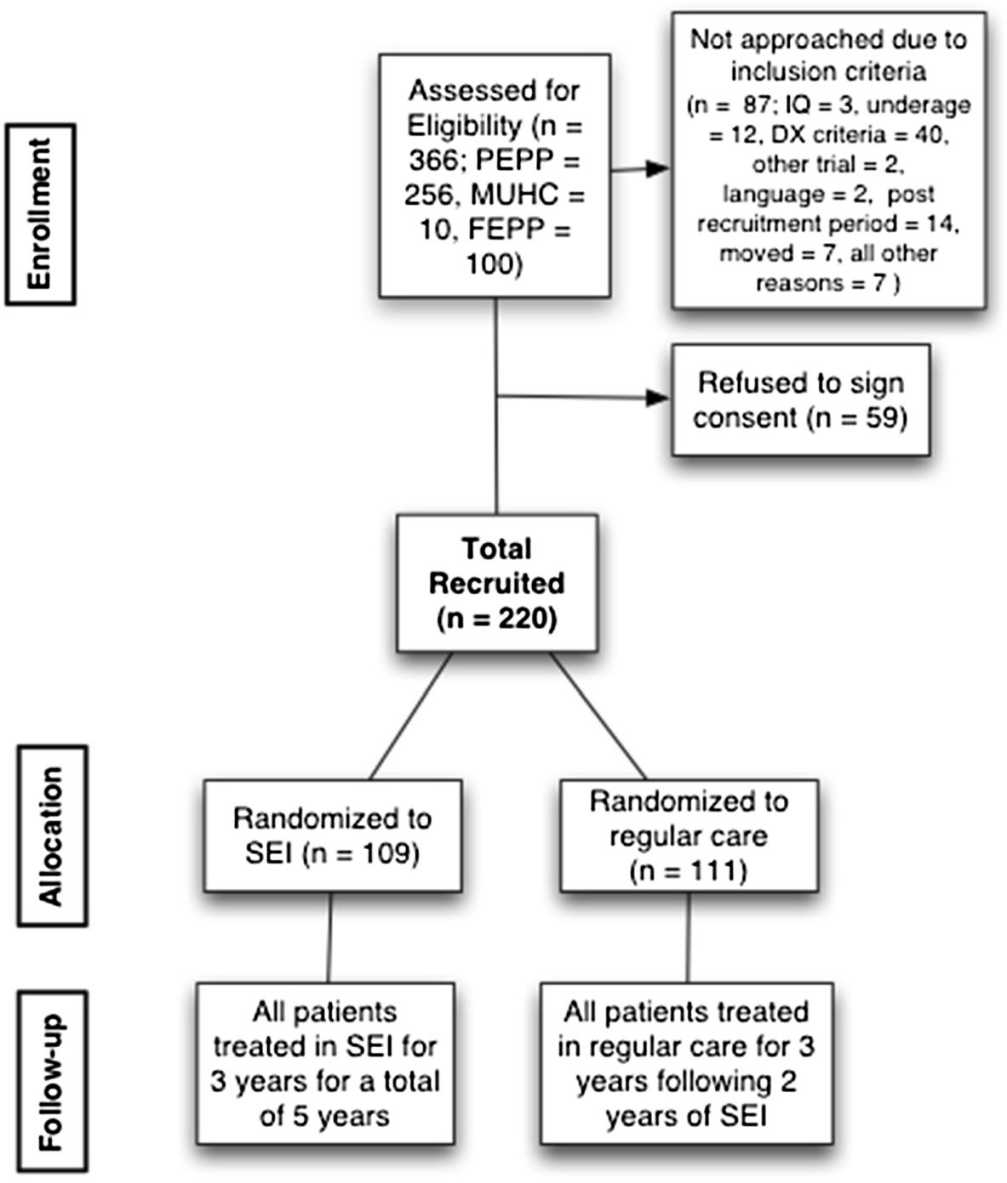
**Diagram of study participants in PEPP trial of extended SEI.**

Randomization is stratified according to sex and substance abuse to ensure that these two factors, know to influence outcome, are balanced between groups. Once participants have signed informed consent to be randomized, their initials and ID # are given to an on site statistician who is not connected with the service. Randomization to one of the two treatment conditions is conducted using a computerized urn randomization protocol [33]. Post-randomization, patients are asked which condition they would have preferred to be randomized to and if they are satisfied with the allocation they were assigned. Results of the randomization are communicated to the treatment team such that appropriate transfer decisions may be made, in the case that a patient is randomized to regular care. This data will be used as a covariate in case that treatment preference biases outcomes (Figure 1).

### Recruitment

Patients who have received ongoing modified case management for the entire two-year period are screened for their participation. Patients that meet the inclusion criteria are approached for their participation between months 21 and 24 of SEI from one of the three McGill network SEI sites. The principal site of recruitment is the Prevention and Early Intervention Program for Psychosis (PEPP-Montreal), established in 2003 and the largest of the three sites; FEP Program at the Jewish General Hospital (established in 2007) and the PEPP at the McGill University Health Centre (established in 2009).

### Participants

#### Blinding

Neither the participants nor case managers and other clinicians can be blinded to the assignment of the treatment condition. However, trained research staff, not involved in the patients’ care and blinded to the treatment condition, conduct all assessments outside any of the patient treatment facilities. Baseline assessments are conducted as soon as possible, within the first 3-6weeks of randomization, in order to avoid revealing group assignment. This is done to prevent any possibility of bias in self-reports that could influence the comparability between groups. In cases where the blind is broken, additional analyses, excluding the unblinded cases, will be conducted to test for any effect of unblinding.

### Interventions

#### The experimental intervention

Patients randomized to three years of extended SEI service continue to have access to the entire package of SEI services for an additional three years, following the initial two years of SEI. The following services, offered at the main PEPP site, constitute the experimental intervention:

- *Modified assertive case management* will continue to be one of the core treatment services provided. The case manager, with professional backgrounds primarily in social work or nursing, who has been involved in the patient’s care in the first two years, continues to provide the same service. This includes supporting the patient towards the attainment of appropriate treatment goals with a moderate case manager to patient ratio (20:1). Goals of treatment typically emphasize adherence to antipsychotic medication, reintegration into employment and/or educational activities, improving patients’ understanding about their illness, reducing dependence on hospital services, providing crisis intervention, promoting independence, monitoring early signs of relapse and reducing the risk of being engulfed by the illness. A personalized profile of the patients’ early warning signs [34,35] is created jointly in collaboration with the patient and used as a tool for the patient and the case manager to monitor any symptoms that might signal future relapse. Case management is provided as per the patients’ needs, using a guideline of a minimum of two contacts per month.
- *Multiple Family Intervention* is offered to the patient’s family as booster sessions of structured family education intervention and multiple family group intervention [36] similar to what is offered during the first two years.
- *Psychoeducation for families* is offered once a year and as booster sessions to the patient’s family. Workshops are designed as three two-hour sessions where families can ask questions and learn about psychosis, treatment and support.
- *Cognitive behaviour therapy* (*CBT*), known to be highly effective for those with psychosis [37,38], is offered in the case of a major depressive episode, anxiety disorder or residual psychotic and/or negative symptoms.
- *Substance abuse education and monitoring* for problems associated with substance abuse is offered to patients who, at initial presentation had a co-morbid diagnosis of substance abuse or developed substance abuse during the first two years of treatment. Therapists have received training to provide a brief (one-two sessions of 40–60 minutes each) intervention based on Motivational Interviewing [39]. The administration of the Timeline Follow-Back procedure followed by feedback [40] is used to help patients track their own alcohol and drug consumption. Patients are referred to appropriate rehabilitation if needed.

#### The control intervention

Patients randomized to the control condition receive treatment as usual in general medical or regular psychiatric services that are available for free to all Quebec patients. Primary regular care in Quebec is predominantly offered through local health and community services centres (CLSCs) that provide health and social services to their catchment area. Care by a family physician in the community is provided in a variety of settings, including at CLSCs and private clinics, and is of variable quality and intensity. Secondary (psychiatric) regular care, including hospital in- and out-patient services, offer a range of psychosocial rehabilitation services, that are part of regular care available to all patients randomized to the control condition.

### Assessments

Evaluations and assessments are carried out at entry and every three months thereafter for the entire follow up period, or until withdrawal from the study, for both treatment conditions (details of assessments are provided in Table 1). If a patient withdraws, they are asked to provide one last assessment. When no contact has been made for 3 consecutive assessment periods (for a total of 9 months) and attempts made for contact through phone calls, emails, and contact with care providers including family members, are not successful, the patient is considered to be withdrawn. Trained evaluators will conduct assessments largely through a semi structured interview format.

**Table 1:** **Assessment instruments**

**Assessment**	**Instrument**
Psychopathology	Scale for the Assessment of Positive Symptoms (SAPS) [41]*
	Scale for the Assessment Negative Symptoms (SANS) [42]*
	Positive and Negative Syndrome Scale (PANSS) [43]*
	Brief Psychiatric Rating Scale (BPRS) [44]*
Functioning	Global Assessment of Functioning Scale (GAF) [45]*
	Social and Occupational Functioning Scale (SOFAS) [46]**
	Productivity Interview Questionnaire (adapted from the Client Socio-Demographic and Service Receipt Inventory) [47]*
	Life Skills Profile [48]**
Medication and side effects	Patient Compliance Interview [49]*
	Medication Compliance: Pill Count Form [49,50]*
Substance use	Chemical Use/Abuse/Dependence-Scale (CUAD) [51]*
	Time Line Follow Back [40]*
Working alliance	Working Alliance Inventory (WAI) [52]**
Randomization preference	Self administered questionnaire***
Quality of life	Life Satisfaction and Psychological Well-being domains of the Wisconsin Quality of Life-Client version [53]**
Duration of untreated psychosis	Circumstances of Onset and Relapse Schedule (CORS) [11]***
Promorbid adjustment	Premorbid Adjustment Scale (PAS) [52]***
Service use including hospital admittance and total bed days	Self administered questionnaire with list of services received
	Regie de l’Assurance-maladie du Quebec (Quebec medical insurance; RAMQ)****
	Med-Echo (for hospitalizations)****

*Assessed at each evaluation (every 3 months).

**Assessed at every second evaluation (every 6 months).

***Assessed once at baseline.

****Assessed post study completion.

Data on the Duration of Untreated Psychosis (DUP) are available for all PEPP patients and is derived using the Circumstances of Onset and Relapse Schedule (CORS) [11], a structured interview instrument for use with patients and families that includes some sections from the Interview for the Retrospective Assessment of Onset of Schizophrenia (IRAOS) [54]. DUP data for all other patients is available through chart verification.

### Outcomes

In light of more recent consensus criteria for remission that emphasizes the amelioration of positive as well as negative symptoms and a return to social and occupational functioning [55], a full range of complementary measures of clinical and functional outcome is being examined both separately and in context with each other.

### Primary outcome

The primary outcome is complete remission measured as both the proportion of patients in remission, as well as the mean length of remission achieved following randomization during the additional three years of follow up. Complete remission is defined according to consensus criteria as a rating of mild or less on the following positive and negative symptom scale items (Positive domain: hallucinations, delusions, bizarre behaviour, positive formal thought disorder; Negative domain: affective flattening or blunting; alogia; avolition-apathy; anhedonia-asociality) for a period of six months [55]. Complete remission is chosen as the primary outcome measure as per findings that remission across both symptom domains is a better predictor of functional outcome than remission of positive symptoms alone [56,57]. Remission is measured at each assessment covering the three months prior using the Scale for Assessment of Positive Symptoms [41] and the Scale for the Assessment of Negative Symptoms [42]. The SANS domain of Attention is not included in SANS ratings as these items have not been shown to correlate to the domain of negative symptoms [58].

### Secondary outcomes

- *Clinical outcome*: (i) Relapse (defined as the reemergence of positive symptoms as measured by a global item on the SAPS of at least 3 in severity that leads to an increase or change in antipsychotic medication or to hospital admission) [59]; (ii) Level of positive and negative symptoms (SAPS [41] and SANS [42] ratings); (iii) Global Assessment of Functioning (GAF) [45]; (iv) Discontinuation of treatment as determined through a lack of service use.
- *Functional outcome* (assessed on two functional dimensions): (i) role functioning (paid employment, school attendance, and/or meaningful housework) and through; (ii) social functioning (such as independence in community living) assessed using the Life Skills Profile [48] as well as the Social and Occupational Functioning Scale (SOFAS) [46] as a global measure of functioning.
- *Quality of Life* (*QOL*): Subjective reporting of QOL is assessed at study entry and subsequently at every six months using the Life Satisfaction and Psychological Well-being domains of the Wisconsin Quality of Life-Client version [53].

### Mediating variables

- *Adherence to medication* is assessed through patient and family reports (when available), and pill counts are conducted by the evaluator. Ratings are made on a five point scale ranging from 0 = not taking medication when prescribed, to 5 = taking medication all of the time as prescribed.
- *Working alliance* with respective service providers is measured with the Working Alliance Inventory-patient version, a self-report instrument [52]
- *Premorbid adjustment* has been shown to be of importance to clinical outcome [11,18] and is assessed with the Premorbid Adjustment Scale [60] at time of entry into the trial in case that such data was not already collected as part of the initial protocol of entry into PEPP-Montreal.

### Economic analysis

Administrative databases are utilized to assess costs associated medical services. The Regie de l’Assurance-maladie du Quebec (RAMQ) provides free public health and prescription drug insurance plans to all eligible Quebec residents. RAMQ databases are consulted for physician services (all patients) and filled prescriptions for patients with public coverage (approximately 90% of patients), and Med-Echo, for hospitalizations. Questionnaires, administered at baseline and every three months following are utilized to record data on any other health care usage as well as for patient and family time involvement in receiving such treatment. We request consent to access this database as part of the initial informed consent. This information is used to track overall societal and economic costs associated with patient treatment in both the experimental and control groups.

### Training and inter-rater reliability

All service care providers and assessors acquire intensive onsite training in their field and are supervised by the principal investigator (A.M), co-PI (R.J) and the PEPP research coordinator (S.A). Assessors have achieved high inter rater reliability (range 0.75-0.92) and are able to consult with the project coordinator should any questions arise.

### Power and sample size

Power Analysis for sample size calculation is based on the proportion of SEI patients that were in remission of positive symptoms in the last two years of the OPUS trial (41.8%) [31] and the proportion of SEI patients that were in remission in the last two years of the uncontrolled outcome study conducted at PEPP–London (69%) [32]. Assuming conservatively a 5% greater proportion of patients in remission for the control condition than in the OPUS trial and a 5% smaller proportion for the experimental condition than in the PEPP-London study, it is estimated that a sample size of 82 in each group (total of 164) will achieve 80% power to detect a difference between groups. Given the rates of attrition between the beginning of the third and the end of the fifth years from the OPUS extension trial that were 18% [31] and from the PEPP-London study that were 12% [32], we expect rates of 25% and <20% over the same period from the control and experimental conditions respectively. Recruitment of participants are adjusted from n =164 to n = 212 to account for possible dropouts.

### Participant withdrawal

For the purpose of this trial, dropouts are considered those with three assessments in a row that are missing. To reduce attrition, our study coordinator contacts anyone who has missed two assessments in a row, prior to their next scheduled evaluation. When a dropout has occurred, an effort is made to ascertain the reason for the dropout and to conduct a final assessment for the primary outcome (remission status). If a patient drops out of treatment but continues to consent to using their data, medical files for their case are located to obtain information on many of the outcome measures.

### Statistical analyses

Data analysis is based on the intent to treat principle. All patient evaluations will be included in the analysis. To assess homogeneity at baseline, relevant demographic data will be presented.

For the primary outcome measures, the proportion of patients in remission in the experimental and control groups will be compared using a Pearson chi-square statistic and the mean length of remission with a t-test or Wilcoxon test for independent samples, based on the distribution of the independent variable. Logistic regression analyses and multiple regression analyses will also be performed with all covariates and mediators.

For the secondary outcome measures including relapse (relapse vs. no relapse), dichotomous outcomes will be analyzed using logistic regression with covariates. Time to Event (relapse) will be measured using a Kaplan-Meier methods and Cox regression to analyze intervals of time from randomization to relapse. Continuous outcomes that include clinical outcomes will be assessed using regression models with covariates and finally longitudinal data with repeated measurements will be analyzed using repeated measures of analysis of variance.

Missing data will be assessed to determine if they are random or informative. Should missing data be non ignorable, then selection models and pattern-mixture models will be used to evaluate the robustness of the primary analyses.

### Ethical considerations

All patients randomized to either continued SEI or to regular care following two years of SEI are offered treatment according to best practice. Participants are informed about the trial and about the voluntary nature of their participation with both written and verbal communications. Participants are only randomized following the provision of informed consent.

## Trial status

Currently all participants (n = 220) have been recruited and are randomized with n = 109 in the experimental group and n = 111 in the control condition (extended SEI vs. regular care). Participants are being followed with the last patient assessments scheduled for 2015.

## Discussion

This study is based on previous data that suggests that the benefits of SEI services in FEP at two years are lost at five-year follow up if patients return to regular care [31]. Given the severity of psychosis and the individual and societal costs associated with this disorder, there is a need for research that can better guide best practices. That FEP patients are young and potentially still malleable to treatment, suggests the importance of positively influencing long-term trajectories of outcome. To our knowledge, this is the first RCT trial of its kind in North America, and one of only two such trials anywhere (OPUS-II) [61], being carried out currently to assess the impact of extended SEI for a total of five years in FEP. Several strengths of this study include the number of participants recruited as well as the computerized randomization procedure that ensures chance allocation to group. That we have included FEP patients with comorbid disorders, including comorbid substance use, and that patients in our control condition are likely to be filtered through a wide range of 1st, 2nd, and 3rd line services available, gives critical construct validity to our study. Given that the majority of service is provided out of a single site (PEPP-Montreal) with two satellite clinics that are closely aligned and that follow the same treatment system, homogeneity of service infrastructure and fidelity to the same SEI model of care is ensured. As well, we are measuring a host of critical variables that may give meaning to the context of remission. Variables including social and occupational functioning and negative symptoms at baseline have been shown to impact long-term trajectories in FEP [12,13]. Measures of quality of life bring into focus the personal perspectives of this disorder while concurrent measures of all associated economic costs will highlight societal burden. This trial is registered http://www.isrctn.com/ (ISRCTN11889976), which helps to ensure complete reporting.

A limitation of any service based trial is that we are unable to blind participants or service providers to treatment allocation. While participants may not receive their preferred allocation in either condition, this possibility is discussed beforehand. That assessors are independent of treatment providers and conduct their evaluations blind to the treatment allocation outside of the patients’ treatment location will reduce chances of unblinding the treatment assignment and ensure the integrity of symptom and functional assessments.

### Potential impact of the results

Despite the growth of SEI services across the world, the optimum length of SEI services in FEPP has not been ascertained and remains an important issue for treatment providers, service users, and policy makers. Results of this RCT are likely to have major impact on treatment of FEP and the recommendation of an optimal duration of SEI services. Should an extension of SEI serve to improve outcomes for patients with a FEP, this will be a major benefit to individuals, families and to society.

## Abbreviations

RCT: Randomized controlled trial
SEI: Specialized early intervention
FEP: First episode psychosis
PEPP: Prevention and early intervention program for psychosis
CLSCs: Community services centres
RAMQ: Regie de’lAssurance-maladie du Quebec
MUHC: McGill University Health Centre
